# Relationship between Citizens’ Health Engagement and Intention to Take the COVID-19 Vaccine in Italy: A Mediation Analysis

**DOI:** 10.3390/vaccines8040576

**Published:** 2020-10-01

**Authors:** Guendalina Graffigna, Lorenzo Palamenghi, Stefania Boccia, Serena Barello

**Affiliations:** 1 EngageMinds HUB–Consumer, Food & Health Engagement Research Center, 20123 Milan, Italy; guendalina.graffigna@unicatt.it (G.G.); serena.barello@unicatt.it (S.B.); 2 Department of Psychology, Università Cattolica del Sacro Cuore, Largo Gemelli 1, 20123 Milan, Italy; 3 Faculty of Agriculture, Food and Environmental Sciences, Università Cattolica del Sacro Cuore, via Milano 24, 26100 Cremona, Italy; 4 Section of Hygiene, Department of Health Sciences and Public Health, Università Cattolica del Sacro Cuore, 00168 Rome, Italy; 5 Department of Woman and Child Health and Public Health, Public Health Area, Fondazione Policlinico Universitario A. Gemelli IRCCS, 00168 Rome, Italy; 6 Faculty of Psychology, Università Cattolica del Sacro Cuore, 20123 Milan, Italy

**Keywords:** vaccine hesitancy, COVID-19, SARS-CoV-2, health engagement, vaccine attitude, path model, mediation model, vaccine acceptance, patient engagement

## Abstract

The actual effectiveness of the still-to-come vaccination against the coronavirus SARS-CoV-2 might be challenged by vaccine hesitancy, a rather common and known phenomenon whose psychological predictors are, nevertheless, still largely debated. Our study aims at understanding how adult citizens’ health engagement, perceived COVID-19 susceptibility and severity, and general vaccine-related attitudes affect the willingness to vaccinate against COVID-19. To that end, on a sample of Italian citizens, we implemented a path model to test the impact of health engagement on the willingness to vaccinate against SARS-CoV-2, and whether this relationship is direct or mediated by the general attitude towards vaccines and the risk perception. Moreover, we tested the configural and weak invariance of the model across gender and three age groups. Results show that health engagement is positively related to the intention to vaccinate and that this relationship is partially mediated by the general attitude towards vaccines. The model appears invariant across genders and partially invariant across age groups, showing some differences in the role of perceived susceptibility. These findings vouch for the implementation of educational campaigns aimed at sustaining future vaccination programs that also include health engagement promotion.

## 1. Introduction

Current efforts towards the development of an effective vaccine against the coronavirus SARS-CoV-2 might be challenged by vaccine hesitancy-which is a difficulty in accepting or an outright refusal of vaccines, despite their availability. Given the current situation, the issue concerning vaccine hesitancy is urgent, but it is not new: in 2019, before the COVID-19 pandemic, the World Health Organization listed vaccine hesitancy as one of the ten global threats to public health [1]. Public health organizations should then prioritize the promotion of the willingness to vaccinate in the whole population. In the recent past, the vaccination rate for the anti-H1N1 vaccine in the 2009 influenza pandemic was below expectations [2], and in this context understanding citizens’ intentions to be vaccinated against COVID-19 might inform policy makers and scientists on educational needs. Several researchers have focused on identifying the potential barriers to vaccine acceptance [3]; among them, Neumann-Boheme et al. [4] surveyed a broad sample of European citizens, and reported that there is a diffused vaccine hesitancy towards the future vaccine against COVID-19, and that it seems to be higher among women and in younger people. Other studies reported that the level of perceived severity of the COVID-19 disease and the level of perceived personal vulnerability to the risk of contagion have impacts on vaccine hesitancy [5,6]. Other studies showed that incorrect health beliefs, conspiracy theories, and general worries for the safety and effectiveness of the future vaccine are important factors that can lead to vaccine hesitancy [7,8]. 

Although the debate about COVID-19 vaccine hesitancy is growing, current reports are mostly focused on circumscribed beliefs and attitudes about the specific vaccination against COVID-19, i.e., beliefs about the effectiveness and the safety of the future vaccine, or the perceived severity of COVID-19, which are addressed as distinct domains. Instead, in our perspective, understanding the interrelatedness of such psycho-social factors in affecting the citizens’ intention to vaccinate against COVID-19 is relevant to shedding light on the complex psychological dynamics which might hamper individual motivation to undertake vaccination, and on that basis, to planning as soon as possible personalized counselling and educational initiatives which may “cultivate” a more positive approach to disease prevention and vaccination behaviors in the target population [9,10,11]. Moreover, previous studies suggested that the level of individual health engagement, defined as individual proactivity in the management of health-related issues, is an important predictor of preventive behaviors [12]. Indeed, the level of individual health engagement can affect their own perceived severity and personal susceptibility to health-related issues by orienting their adherence towards preventive behaviors [13]. This fact has been demonstrated in the context of the COVID-19 pandemic, as health engagement has been shown to be a good predictor of individual preventive attitudes [14,15,16]. More recently, research has called for further exploration of the concept of health engagement in the context of vaccine-related behaviors, which can be considered a health behavior, as it entails the immunization of a healthy person against an infectious disease [17]. 

To fill this knowledge gap, this study aimed at surveying Italian adult citizens to get insights into how health engagement, perceived COVID-19 susceptibility and severity, and general attitudes towards vaccines affect their willingness to vaccinate against COVID-19 and whether these variables play different roles across gender and age groups. 

## 2. Materials and Methods 

### 2.1. Study Design and Participants

A random sample (*n* = 1004) of Italian adult citizens was asked to complete a survey during the early days of the Italian reopening after the lockdown (i.e., phase 2). The survey concerned the impacts of the COVID-19 pandemic on their lives and health management habits. A specific section of the survey was dedicated to exploring participants’ beliefs and attitudes towards the future vaccine against COVID-19. A professional panel provider (Norstat-Italia srl), using a stratified sampling strategy, was asked to recruit the random sample across all Italian regions. Inclusion criteria were being able to read and understand Italian language, living in Italy, and being at least 18 years old. 

### 2.2. Measures

The survey involved a series of questions used to profile the sample from a socio-demographic point of view. 

Moreover, participants were asked to answer 6 ad-hoc items regarding their health engagement (HE) on a 5-point Likert scale; answers were then averaged to compute a general score of HE ranging from 1, low HE, to 5, high HE. Participants were also asked two questions regarding their vaccine attitudes (VAs), which were then averaged to compute a single score for each participant ranging from 1, negative attitude, to 5, positive attitude. Finally, participants were asked two things regarding the perceived severity of and susceptibility to COVID-19 (answered on 10 and 5-point Likert scales, respectively), and a single thing regarding their willingness to vaccinate against COVID-19 whenever the vaccine is available (answered on a 5-point Likert scale where 1 is low probability to vaccinate and 5 is high probability to vaccine).

The questionnaire’s items are available in the appendix translated into English (Supplementary Materials file 1).

### 2.3. Statistical Analyses

Descriptive statistics such as means, standard deviations, skewness, and kurtosis were computed to describe our sample and assess variables distribution. In particular, skewness and kurtosis were assessed to check for normal distribution: a parameter outside the −1/+1 range is generally considered an index of non-normality, and as a matter of fact, different methods of estimation should be used for path analysis. 

Moreover, in order to assess the reliability of the summed scores, Pearson’s correlations were applicable, and Cronbach’s α was computed: in particular, we considered it acceptable to compute a summative score whenever there was either a moderate correlation index (>0.40) or a Cronbach’s α index above the 0.70 threshold. These analyses were carried out with IBM SPSS v23.

In order to test the hypothesized relationships between variables included in the theoretical model, a path analysis was carried out using the Lavaan package (v0.6–6) [18] based on R version 4.0.1. Path models are a statistical method that, compared to multiple regressions, allow for the simultaneous assessment of several regression paths occurring between multiple dependent and independent variables and for the computing of direct, indirect (mediated), and total effects. 

In the path analysis, the goodness of fit of the hypothesized model is usually assessed by calculating a series of indices; the more commonly used indices are: a non-significant χ^2^, and for bigger sample sizes, a χ^2^/df ratio <5 is considered an index of good fit [19]; root-mean-square error of approximation (RMSEA) is generally considered acceptable when <0.080 [20]; standardized root mean square residual (SRMR) is considered acceptable below the 0.080 threshold as well; and finally, comparative fit index (CFI) should be above 0.95 [21]. Model parameters and standard errors estimates were calculated with MLM estimator, which allows a robust estimation of standard errors and Satorra–Bentler corrected test statistics [22].

Finally, we tested the model structural invariance across the relevant groups (i.e., gender and three age groups, namely, 18–38, 39–52, >52; cut-offs were decided according to the 33rd and 66th percentiles, respectively). Invariance is generally tested in subsequent steps, by adding constraints to the model and by comparing the fit indices of the nested model with the former [23,24]. 

In particular, the four subsequent steps are:

- Configural invariance: is the model’s structure equal across groups?
- Weak invariance: are regressions between variables equal across groups?
- Strong invariance: are intercepts equal across groups?
- Strict invariance: are residuals equal across groups?

The model with more constraints is considered acceptable if its fit indices do not deviate too much when compared to the more parsimonious model (in particular, it is considered acceptable if the difference between chi-squares is not significant [25], if the difference between CFI is <0.01 [26], if difference in RMSEA is <0.015, and if SRMR difference is <0.030 [27]). Since it is not a nested model, the configural invariance model is instead evaluated based on its absolute fit indices.

Nevertheless, we only tested configural and weak invariance, as we were interested in investigating possible differences in the relationships between the variables rather than demonstrating a complete invariance.

Raw data and lavaan scripts for the path analysis and the invariance analysis have been made available as electronic supplementary materials (see Supplementary Spreadsheet S1: raw data and R-script S2: lavaan syntax, respectively).

### 2.4. Ethics

This study is part of a broader project aimed at exploring citizens’ lives, health habits, and food consumption. Each participant was informed about the aims of the study and provided informed consent before filling in the questionnaire. Participants were allowed to drop out at any time. The study has been performed in accordance with the Declaration of Helsinki and was approved by an independent ethical commission of the Department of Psychology, Università Cattolica del Sacro Cuore, Milan (Italy) (CERPS—IRB#02–20).

## 3. Results

### 3.1. Sample Characteristics

Table 1 shows the sample characteristics. The sample was composed of 1004 Italian adult citizens (50.9% female). The average age was 44 with a standard deviation of 14, ranging between 18 and 70. Among the whole sample, 15.3% answered that the probability of them getting the vaccine was either “not likely at all” or “unlikely”, and 26.2% answered that it is “not likely or unlikely.” Only 58.6% answered that they would probably accept the vaccine.

### 3.2. Descriptive Statistics and Reliability

As Table 2 shows, most of the variables used for the model had rather low skewness and kurtosis, hence vouching for a normal distribution. Nevertheless, since one variable showed values of both skewness and kurtosis outside the −1/+1 rule of thumb, we preferred a robust estimator (MLM, as already discussed).

Pearson’s test of correlation revealed that the two items regarding the attitude towards vaccines were indeed correlated (r = −0.39; *p* < 0.001); since the index is very close to the threshold of 0.40, we found it acceptable to compute a single score from these items. Since correlation was negative, item 1 was reversed.

Finally, Cronbach’s α for the health engagement items was 0.75, which vouches for an acceptable internal reliability and for computing a single score.

### 3.3. Path Analysis

The fit indices showed that the hypothesized model had an acceptable fit with data. In particular: robust χ^2^(df = 2, *n* = 1004) = 7.255, *p* = 0.027; χ^2^/df = 3.63; CFI = 0.99; SRMR = 0.021; and RMSEA = 0.063 (LO90 = 0.028; HI90 = 0.104). The confidence interval of RMSEA had a higher bound above the generally accepted threshold of 0.080; nevertheless, this was probably due to the tested model having only two degrees of freedom, as it is known that this index tends to indicate poor fits in such models [28]. Overall, the model was acceptable. 

Parameter estimates showed that there was a significant, strong positive relationship between the general attitude towards a vaccine and the intention to vaccine against COVID-19 (std. β = 0.651; *p* < 0.001). Moreover, there was also a significant, positive relationship among perceived severity, perceived susceptibility, and willingness to vaccinate (std. β = 0.132; *p* < 0.001 and std. β = 0.085; *p* = 0.005, respectively). Finally, results showed that there was also a significant, positive direct relationship between health engagement and willingness to vaccinate, though a modest one (std. β = 0.080; *p* < 0.001).

However, as Table 3 shows, the relationship between these variables was partially mediated: in particular, there was a marginally significant indirect path passing through susceptibility (std. β = 0.009; *p* = 0.039), a significant, though modest, indirect path passing through severity (std. β = 0.024; *p* = 0.001), and a significant indirect path passing through the general attitude (std. β = 0.074; *p* < 0.001). Overall, the indirect effects accounted for most of the total effect of health engagement on willingness to vaccinate (0.108/0.188).

**Figure 1:**
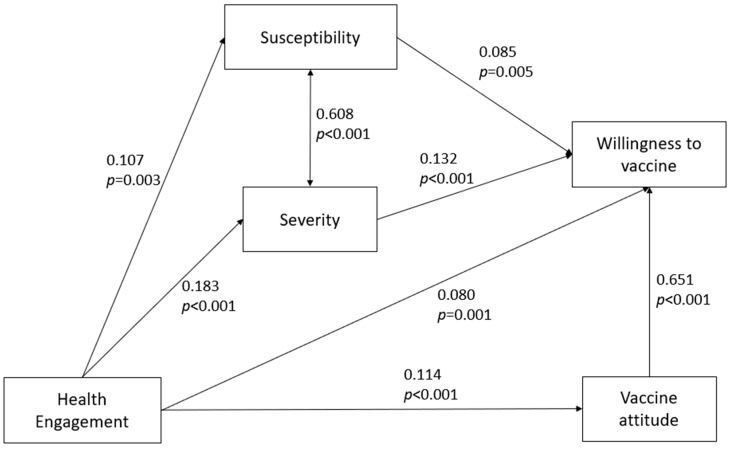
Tested path model. Numbers on arrows represent standardized regression weights and relative *p*-values.

Figure 1 shows the tested path model with standardized regression estimates and relative *p*-values.

### 3.4. Invariance Analysis

Table 4. All the steps and fit indices of the invariance analysis.

The fit indices of the model split by gender (configural invariance) seemed acceptable: robust χ^2^_(df = 4, *n* = 1004)_ = 10.533, *p* = 0.032; χ^2^/df = 2.63; CFI = 0.99; SRMR = 0.026; and RMSEA = 0.072 (LO90 = 0.035; HI90 = 0.114). Testing the invariance of regression weights (weak invariance) did not seem to deter model fit significantly: Δχ^2^ = 7.607_(8)_, *p* = 0.47; ΔCFI = 0.00; ΔRMSEA = −0.028; ΔSRMR = 0.012. The fit indices of the model split by age groups also seemed acceptable: robust χ^2^_(df = 6, *n* = 1004)_ = 12.015, *p* = 0.062; χ^2^/df = 2.00; CFI = 0.99; SRMR = 0.030; and RMSEA = 0.071 (LO90 = 0.031; HI90 = 0.114). However, testing the invariance of regression weights (weak invariance) for age groups caused a significantly lower model fit for the nested model—Δχ^2^ = 29.232_(16)_, *p* = 0.022; ΔCFI = ‒0.015; ΔRMSEA = −0.009; ΔSRMR = 0.028—implying that at least for one group there was a significantly different regression weight. By inspecting regression weights, we tried to test a partial weak invariance by removing constraints on the estimates that appeared more divergent than those of the other groups, namely, the regression from susceptibility to willingness to vaccinate for the middle-aged group. As results show, fit indices of the new nested model were compatible with a partial weak invariance: Δχ^2^ = 16.204_(15)_, *p* = 0.368; ΔCFI = −0.003; ΔRMSEA = −0.026; ΔSRMR = 0.017.

Table 5 reports regressions by age groups along with direct, indirect, and total effects of health engagement on willingness to vaccinate for comparison.

## 4. Discussion

While scientists around the world are committed to the development of a new vaccine against COVID-19, the vaccine hesitancy of the population can constitute one of the main hindrances for a future effective immunization against COVID-19. Indeed, in our sample about 15% stated that they would probably refuse the vaccine, while another 26% would be hesitant. This is in line with other survey data from western countries: in France, 26% of the respondents stated that they would not use the vaccine [29]; in the US, 20% would decline [30]; in Poland, 28% [31]. The same authors that polled the vaccine hesitancy in Poland also reported the results from a systematic search of other national surveys, finding that while there is a high variability in the number of hesitant people (from very low, such as in China, 2%, or UK, 4%, to very high, such as in Turkey, 44%, or Czech Republic, 43%), among the 20 nations polled in their study, most of them would not reach the 67% necessary for hitting the herd immunity threshold. Moreover, and worryingly, the level of unwillingness to vaccinate against COVID-19 was higher than vaccination reluctance for usual vaccines in several countries [31]. 

These findings not only show how vaccine hesitancy is a diffused, world-wide problem, but also point out that there could even be different and specific concerns regarding the future COVID-19 vaccine, and that studying and understanding social, demographic, and psychological determinants is fundamental for the success of the future national immunization plans, in order to better understand which groups in the population are more likely to refuse the vaccine, and on the other hand, which psychological levers can be used to increase willingness to vaccinate.

Our study suggested two major pathways between health engagement and intention to vaccinate against COVID-19: a direct path between health engagement and intention to vaccinate, and an indirect one that is mediated by the general attitude towards vaccination. The model is independent of gender and age group. 

The perceived susceptibility towards COVID-19 and disease severity were marginally relevant both as predictors and as mediating variables. However, in the middle-aged group, the perceived susceptibility seemed to be a more impactful factor. Lastly, an inspection of direct, indirect, and total effects of health engagement on willingness to vaccinate across age groups suggested that among younger people, the relationship between these two variables seems to be proportionally more affected by the direct effect, while in the elderly people the effect is more strongly mediated by the attitude towards vaccination. 

A number of studies are attempting to disentangle the reasons behind vaccine hesitancy in order to timely plan educational campaigns that will sustain and enable an effective vaccination plan by increasing the numbers of adherent persons. The majority of the studies conducted so far showed the roles of socio-demographic variables (i.e., age, gender, geographical area of residence, presence of clinical conditions, etc.) in determining vaccination intention [4]. Previous studies also demonstrated the roles of individual beliefs about the disease’s severity and their own perceived vulnerability to contracting the disease in determining vaccine intention [5,6], along with beliefs about the effectiveness and safety of the vaccine [7,8]. In this study—by adopting a psychological lens—we demonstrated the interrelated relationship among these variables. Moreover, we demonstrated the predictive role of the individual’s general health engagement attitude in the intention to vaccinate against COVID-19. Our results corroborate the relevance of studying the general individual’s psychological attitude towards their health promotion and self-management—namely, health engagement—as a relevant factor impacting on preventive behaviors [15,32,33,34]. Furthermore, these data corroborate the value of extending the current debate about patient engagement to the specific scope of vaccine intention [35]. Finally, these data suggest the opportunity to advance the understanding of psychological factors impacting on COVID-19 vaccine hesitancy by also including the analysis of less contingent elements (i.e., not only the individual’s current state of worry and perceived susceptibility to the disease), such as the individual’s health engagement attitude: a psychological dimension that could be modified thanks to dedicated educational and counselling interventions. Additionally, this result is interesting and may have important implications for public health practice, as young people have been demonstrated to be the most hesitant age group for the future vaccine against COVID-19 [4,6], while also being affected by an “invulnerability bias,” which leads them to feel less at risk from infectious diseases [36], confirming previous studies on this topic [37]. How to improve vaccination behaviors in the young generation is still a topic which is open for debate in different infectious conditions [38,39]. These data suggest that the health engagement attitude is predictive of COVID-19 vaccination intentions among younger people as well, and that this relationship is, mostly, not mediated by other variables. This may imply the need to foster not only vaccine-specific attitudes, but also general positive attitudes towards health and health self-management among the youngest generations, which in turn encompasses different preventive conduct [40].

This evidence is important because it suggests how the intention to vaccinate is framed by psychological determinants and may be the result of an attitudinal change in one’s own health management: making the population perceive them as proactive in self-management and health promotion may improve individual acceptance towards a new vaccine, such as the one against COVID-19. These claims for timely actions are aimed at fostering a more effective psychological attitude towards health in the population starting from school age. The goal is to make individuals perceive themselves as co-responsible for their own health. Furthermore, educating people to become more engaged in prevention may be the basis for nourishing a positive climate of partnership and collaboration between the healthcare system and its users, in all the different phases of the life cycle. Clinical and organizational evidence about the value of engaging patients in healthcare is growing. Our study suggests broadening this scientific debate to vaccine-related attitudes as well, since it shows the role of engagement in promoting COVID-19 vaccine acceptance. Furthermore, our study unveils the psychological factors which sustain individual orientation towards health engagement. This may reveal a new important scenario for the development and implementation of future educational campaigns to sustain an effective vaccination program.

## 5. Conclusion and Limitations

This study has some limitations, and results should be interpreted and used with caution. Firstly, the measures used in this study were self-reported and might be subject to reporting bias. In addition, the current study adopted a series of measures that were not validated—even if internal consistency was adequate. Moreover, this study refers to a sample of citizens who did not receive the COVID-19 vaccine, as it is not yet available, hence forcibly focusing on intentions rather than on behaviors. However, this is the first time, as far as we know, that health engagement levels have been examined as possible determinants of citizens’ vaccination decision-making.

Second, as an observational cross-sectional study, causal relationships could not be inferred. 

Third, even though the path model had an acceptable fit, regression weights and estimates of indirect and total effects suggest that these results should be taken with caution, as effect sizes were generally modest. Future studies may address these issues by investigating the same hypotheses with validated measures, and when the COVID-19 vaccine will be ready and available for the population, focusing on actual behaviors rather than intentions: this will allow more reliable results, and potentially, more accurate estimates of effects and effect sizes. 

Finally, there are indeed some socio-demographic variables that were not taken into account in this paper, and that is worth discussing. For instance, the presence of chronic conditions such as diabetes or hypertension [41,42] can have an important impact on the clinical outcome of COVID-19, warranting particular attention to the immunization of such groups. Future studies should address this gap by exploring the relevance of vaccine hesitancy in high-risk groups and by investigating eventual differences in both socio-demographic and psychological determinants of hesitancy in these groups.

Regardless of these limitations, to the best of our knowledge, no other study has explored the role of health engagement in effecting vaccine acceptance. Our findings may serve as preliminary considerations, since determinants of hesitancy have been shown in previous studies to be country and context-specific [43]. However, although preliminary, the study findings contributed to confirm the multiplicity and complexity of vaccine hesitancy determinants. Thus, we argue that future studies and interventions on this issue should be based on multifactorial health behavior models, including the promotion of a psychological commitment to health engagement.

## Figures and Tables

**Table 1:** Sample characteristics.

Variable Sub Variable	(*n*)%
Gender	
Male	49.1 (*n* = 493)
Female	50.9 (*n* = 511)
Age group	
18–38	34.4 (*n* = 345)
39–52	33.6 (*n* = 337)
>52	32.1 (*n* = 322)
Geographical region	
North-west	26.3 (*n* = 264)
North-east	18.6 (*n* = 187)
Center	19.7 (*n* = 198)
South and islands	35.4 (*n* = 355)
Education	
No high school	12.5 (*n* = 126)
High school	60.0 (*n* = 602)
University or higher	27.5 (*n* = 276)
Willingness to vaccinate	
Not likely at all	8.6 (*n* = 86)
Unlikely	6.7 (*n* = 67)
Not likely or unlikely	26.2 (*n* = 263)
Very likely	33.3 (*n* = 334)
Absolutely likely	25.3 (*n* = 254)

**Table 2:** Descriptive statistics.

Variable Name	Mean	Standard Deviation	Skewness	Kurtosis
Health Engagement	3.62	0.59	−0.031	0.221
Vaccine attitude	3.37	0.89	−0.220	0.218
Perceived Severity	7.51	2.06	−1.076	1.173
Perceived Susceptibility	3.08	0.96	−0.174	−0.230
Willingness to vaccinate	3.60	1.18	−0.684	−0.229

**Table 3:** Direct, indirect, and total effect of health engagement on willingness to vaccinate.

Path	Std. Estimate	*p*-Value
Health engagement -> Willingness to vaccinate	0.080	<0.001
Health engagement -> Susceptibility -> Willingness to vaccinate	0.009	0.039
Health engagement -> Severity -> Willingness to vaccinate	0.024	0.001
Health engagement -> Attitude towards vaccine -> Willingness to vaccinate	0.074	<0.001
Total health engagement effect (indirect effect + direct effect)	0.188	<0.001

**Table 4:** Model structural invariance and fit indexes.

Gender			Age		
Parameters	Configural	Weak	Configural	Weak	Weak Partial ^2^
Robust χ^2^_(df)_	10.533_(4)_	18.854_(12)_	12.015_(6)_	41.365_(22)_	28.875_(21)_
*p*-value	0.032	0.092	0.062	0.007	0.177
RMSEA (90% C.I.)	0.072 (0.035–0.114)	0.044 (0.016–0.070)	0.071 (0.031–0.114)	0.062 (0.039–0.085)	0.045 (0.015–0.070)
CFI	0.991	0.991	0.992	0.977	0.989
SRMR	0.026	0.038	0.03	0.058	0.047
Δχ^2^_(Δdf)_^1^		7.607_(8)_		29.232_(16)_	16.204_(15)_
*p*-value		0.47		0.022	0.368
ΔCFI				−0.015	−0.003
ΔRMSEA		−0.028		−0.009	−0.026
ΔSRMR		0.012		0.028	0.017

RMSEA: root mean square error of approximation; CFI: comparative fit index; SRMR: standardized root mean residual. ^1^ Scaled χ^2^ difference. ^2^ Removed the constraint on the regression between susceptibility and willingness to vaccinate for the middle-aged group.

**Table 5:** Regression weights and effects of engagement on willingness to vaccinate by age group.

Path	Younger		Middle Aged		Elderly	
	Std. Estimate	*p*-Value	Std. Estimate	*p*-Value	Std. Estimate	*p*-Value
Susceptibility <-> Severity	0.583	<0.001	0.644	<0.001	0.595	<0.001
Health Engagement -> Susceptibility	0.127	0.028	0.108	0.101	0.081	0.137
Health Engagement -> Severity	0.194	0.003	0.170	0.009	0.161	0.003
Health Engagement -> Attitude towards vaccine	0.114	0.042	0.067	0.242	0.190	0.002
Susceptibility -> Willingness to vaccinate	0.010	0.844	0.201	<0.001	0.029	0.586
Severity -> Willingness to vaccinate	0.096	0.075	0.140	0.016	0.196	<0.001
Attitude towards vaccine -> Willingness to vaccinate	0.621	<0.001	0.608	<0.001	0.703	<0.001
Health Engagement -> Willingness to vaccinate	0.134	0.002	0.072	0.073	0.032	0.369
Health Engagement -> Susceptibility -> Willingness to vaccinate	0.001	0.845	0.022	0.133	0.002	0.601
Health Engagement -> Severity -> Willingness to vaccinate	0.019	0.108	0.024	0.057	0.013	0.032
Health Engagement -> Attitude towards vaccine -> Willingness to vaccinate	0.071	0.044	0.041	0.240	0.134	0.002
Total effect of Health Engagement on Willingness to vaccinate	0.244	<0.001	0.158	0.010	0.200	<0.001

## Supplementary Materials

The following are available online at https://www.mdpi.com/2076-393X/8/4/576/s1. Spreadsheet S1: raw data; R-script S2: lavaan syntax; File 1: translated survey.
